# A Missense Mutation rs781536408 (c.2395G>A) of *TYK2* Affects Splicing and Causes Skipping of Exon18 *in vivo*

**DOI:** 10.3389/fgene.2021.679678

**Published:** 2021-06-21

**Authors:** Suqing Chen, Peilin Wu, Bin Wu, Chenye Lin, Junhong Chen, Lishengdan Chen, Ge Lv

**Affiliations:** ^1^Department of Pediatrics, The First Affiliated Hospital of Fujian Medical University, Fuzhou, China; ^2^Chongqing Key Laboratory of Child Infectious and Immunity, Children’s Hospital of Chongqing Medical University, Chongqing, China

**Keywords:** *TYK2*, mutation, splicing, minigene, mRNA

## Abstract

*TYK2* variants can impact disease onset or progression. In our previous study, we identified abnormal splicing that happened near rs781536408 in the *TYK2* gene. The purpose of this research was to examine the effect of the mutation on alternative splicing *in vivo* and *in vitro*. Whole exome sequencing was performed to identify the mutations followed by bidirectional Sanger sequencing. Then the minigene analysis was carried out based on HeLa and HEK293T cell lines. The results showed that rs781536408 (c.2395G>A, p.G799R) was homozygous in the patient, but heterozygous in parents. PCR amplification confirmed the abnormal splicing in the somatic cells of the patients, but not in the parents. Sanger sequencing results showed that there was a skipping of exon18 near the mutation. For minigene analysis, there was no difference between the wild-type and the mutant type in the two minigene construction strategies, indicating that mutation c.2395G>A had no effect on splicing *in vitro*. Combining the results of *in vivo*, we speculated that the effect of the mutation on splicing was not absolute, but rather in degree.

## Introduction

RNA splicing is a process that enables a pre-mRNA to mature mRNA by removing introns and joining together exons. Alternative splicing can be induced by single-nucleotide polymorphism (SNP). Most genes in human beings produce multiple transcript variants due to alternative splicing (Modrek and Lee, 2002). However, the correct pattern of splicing does not always happen. Some splicing errors may lead to diseases (Fernandez Alanis et al., 2012), such as growth hormone deficiency (GHD), spinal muscular atrophy (SMA), and neurodegeneration. There are many reasons for abnormal splicing, among which SNP mutation is an important one. As previously reported, mutations in exon splicing enhancers could induce abnormal transcript formation, affect protein coding, and further make people sick (Blencowe, 2000; Graveley, 2000).

*TYK2* is one of the JAK protein tyrosine kinase family members. *TKY2* mediates the signaling of many antivirals and immunoregulatory cytokines, such as type I and type III IFNs, IL-10, IL-12, IL-22, and IL-23 (Li et al., 2020). However, the effects of *TYK2* mutation on the pathogenesis or progression of the diseases are still in suspense. On the *TYK2* locus, there were over 500 SNPs that can cause non-synonymous encoding. Among them, rs781536408 (c.2395G>A) is a lower-frequency mutation. Besides our previous report (Wu et al., 2020), no other studies have reported on this mutation. As a non-synonymous mutation, rs781536408 may change the structure of Tyk2 protein and may even inactivate Tyk2 protein. For its function, more work is needed for verification.

Single-nucleotide polymorphisms can affect mRNA splicing, nucleocytoplasmic export, RNA stability, and translation. When SNP changed the codon and causes amino acids to change, the protein activity may even be affected (Rodríguez-Martínez et al., 2020).

In this study, we found that the *TYK2* gene can undergo abnormal splicing near rs781536408. So we hypothesized that rs781536408 might be a splicing-relevant mutation. This study aimed to explore the effect of the mutation on alternative splicing *in vivo* and *in vitro*. Therefore, we attempt to clarify the potential molecular mechanism of the relationship between *TYK2* transcripts and the rs781536408 mutation.

## Materials and Methods

### Clinical Examination and Sample Collection

This present study was licensed by the Ethics Committee of The First Affiliated Hospital of Fujian Medical University [ID: MRCTA, ECFAH of FMU (2019)218]. Written informed consent was obtained from all participants. Blood samples were sampled and stored at −80°C. The extraction and purification of DNA and RNA were carried out using an ALLPrep DNA/RNA/miRNA Universal Kit (Qiagen, Hilden, Germany) following the manufacturer’s instructions. The quality control of the purified nucleic acid was performed using the ND-1000 NanoDrop (Thermo Fisher Scientific, Wilmington, DE, United States).

### Whole Exome Sequencing and Analysis

Firstly, genomic DNA was randomly sonicated to generate 150–200-bp products. Then, the sequencing libraries were prepared using the Lybay DNA Library Prep Kit for Illumia (Lybaybio, Tianjin, China). DNA libraries were hybridized to obtain the exon sequence using Lybay Human exon capture Kit (Lybaybio, Tianjin, China). The whole exome sequencing data were generated, processed, and analyzed by Allwegene Technology Co., Ltd. (Beijing, China). The libraries were sequenced by an Illumina HiSeq4000 platform under the paired-end 150-bp mode. The data were filtered using FastQC (version 0.11.9) with default parameters. We aligned the sequenced reads to the human reference genome (GRCh37/hg 19) by Burrows-Wheeler Aligner (BWA, version 0.6.2) and filtered the duplicates using SAMtools (version 1.2), by analyzing the type of mutation sites in the family and comparing and screening the mutation sites that are most likely to cause disease based on the dbSNP, 1000 Genomes Project, and ExAC databases.

### Sanger Sequencing

The screening for *TYK2* mutation was conducted by bidirectional Sanger sequencing. Gene-specific PCR primers were designed and used to amplify sequences applying standard PCR amplification protocols. Briefly, the PCR products were amplificated by ExoSAP-IT method (USB, Cleveland, OH, United States). Then, the products were sequenced using Big Dye Termination Chemistry [Applied Biosystems (ABI), Weiterstadt, Germany] and separated on a DNA capillary sequencer (ABI 3100 genetic analyzer; ABI, Weiterstadt, Germany).

### Plasmid Construction

The minigene construct was generated by gene synthesis (Sangon, Shanghai, China). Two plasmids, pEGFP-C1 and pcMINI-N, were used in the experiment. The mutant (mut) and wild-type (WT) were reconstructed with pEGFP-C1 and pcMINI-N. The reaction system is similar to that the previous report (Rodríguez-Martínez et al., 2020). The recombinant vectors were transiently transfected into HeLa and HEK293T cell lines (American Type Culture Collection, Manassas, VA, United States). The transfection method was carried out according to the liposome 3000 (Thermo Fisher Scientific, Waltham, MA, United States) following the manufacturer’s instruction, and cell samples were collected at the 48th hour. Then, the total RNA in these cells was extracted and the primers on both sides of minigene was used to carry out PCR amplification, using agarose gel to detect the gene transcription band size. The clones’ sequence was confirmed by Sanger sequencing. Primers used for cloning and final DNA sequences are listed in Supplementary Table 1.

### Splicing Prediction

Splicing prediction of WT and mut sequences was conducted using Human Splicing Finder (HSF^1^), MutationTaster^2^, SpliceAI^3^, Spliceogen^4^, and MMSplice^5^ with the Default parameters.

### Quantitative Real-time PCR Analysis

The expression of transcript was quantified by qRT-PCR using a TransScript Green two-step qRT-PCR Supermix kit (TransGen Biotech, Beijing, China) according to the manufacturer’s instructions. Expression of the housekeeping gene β-acting was used as an internal control for normalization. The PCR amplification cycle conditions were 50°C for 2 min, 94°C for 10 min, 95°C for 15 s, and 60°C for 1 min for 40 cycles. The relative expression of the target gene was determined using the Ct (2^–ΔΔCT^) method. The sequences of the primers are listed in Supplementary Table 1.

## Results

### Identification of c.2395G>A Mutation in TYK2 Gene

**FIGURE 1:**
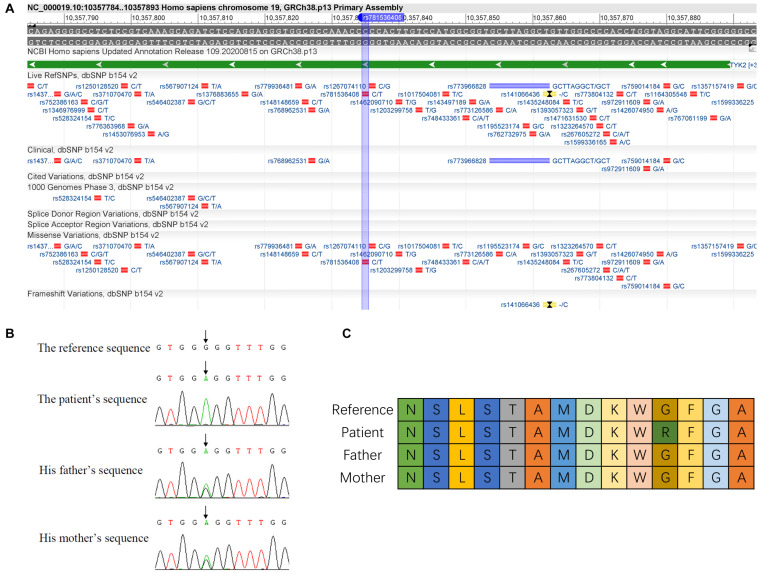
Identification of c.2395G>A mutation in *TYK2* gene. **(A)** Details of c.2395G>A mutation are in dbSNP database (https://www.ncbi.nlm.nih.gov/variation/view/?q=rs781536408www.ncbi.nlm.nih.gov/variation/view/?q=rs781536408). **(B)** Sanger sequencing results of the family. **(C)** Variation of amino acid sequence caused by c.2395G>A mutation (differently colored boxes represent different amino acids).

From the exome sequencing data of all the three family members recruited in the study, only one homozygous mutation was identified in the patient (this mutation is heterozygous in the patient’s parents). This homozygous mutation p.G799R (c.2395G>A) was identified in the *TYK2* gene in the patient (Figure 1A). This mutation (rs781536408) was also reported in GnomAD, TOPMED, and ExAC databases with a frequency of 0.000020, 0.000016, and 0.000009, respectively. The mutation was confirmed in all the family members by Sanger sequencing (Figure 1B). By analyzing the amino acid sequence changes caused by p.G799R mutations in different species, we found that the mutated glycine residue in p.G799R was not evolutionarily conserved (Figure 1C). The four bases upstream (5′) of the mutation site are GTGG, which may be a variable splicing recognition site. The clinical features and phenotypes of the patient showed symptoms such as pneumonia, enteritis, and autism [the case report has been published with the consent of his family (Wu et al., 2020)].

### Bioinformatics Prediction of Variable Splicing Site Caused by c.2395G>A Mutation

The pathogenicity of the c.2395G>A mutation is supported by *in silico* analyses using two different programs: HSF (see text footnote 1) and MutationTaster (see text footnote 2). HSF prediction results showed that the c.2395G>A mutation led to the activation of a new intron splice acceptor site, and the sequence of the intron–exon boundary is ggacaagtggagGT, which might produce three exon splice suppressors (ESSs). MutationTaster predicted that a new donor site was formed, and its exon–intron boundary sequence was ACAA-gtgg. Also, the prediction information of the other databases also confirmed it. The details of the prediction results of SpliceAI, Spliceogen, and MMSplice are shown in Supplementary Table 2.

### Transcription Experimental Analysis Revealed a Different Splicing Pattern Near the Genomic Location of TYK2 Mutation

**FIGURE 2:**
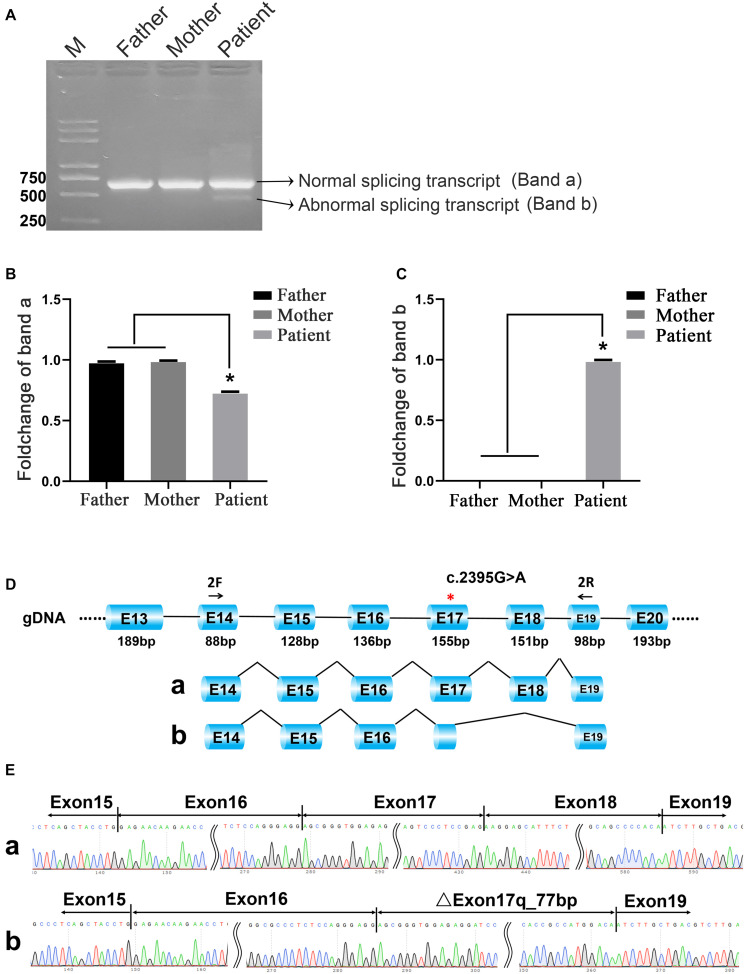
C.2395G>A mutation caused different splicing patterns in the family. **(A)** Agarose gel electrophoresis. Band a and band b represent normal splicing and abnormal splicing, respectively. M means the DNA marker. **(B)** The qRT-PCR results of band a; *p < 0.05. **(C)** The qRT-PCR results of band b; *p < 0.05. **(D,E)** The schematic representation and the Sanger sequencing results of the two different transcripts.

To compare the transcriptional differences between heterozygous and homozygous mutations, RT-PCR followed by Sanger sequencing was performed. The agarose gel electrophoresis of PCR products results showed that both parents of the patient had only one PCR band, while the patient had two (Figure 2A). In order to improve the detection sensitivity, we designed two pairs of specific primers according to the sequence of band a and band b for qRT-PCR detection (primers a and b correspond to band a and band b, respectively). The expression of band a in the patient was about 70% of that in the parents (Figure 2B). In addition, the signal of band b was only found in the patient, not in the parents (Figure 2C). The Sanger sequencing results confirmed that there was only a normal splicing pattern found in the parents [Exon14 (47 bp)–Exon15 (128 bp)–Exon16 (136 bp)–Exon17 (155 bp)–Exon18 (151 bp)–Exon19 (65 bp)]. However, in addition to the normal form of splicing, a novel variable splice form was found in the patient [Exon14 (47 bp)–Exon15 (128 bp)–Exon16 (136 bp)–ΔExon17 (78 bp)–Exon19 (65 bp); Figures 2D,E]. The Sanger sequencing results showed that the novel alternative splicing occurred at the fourth base upstream (5′) of the mutation site with the exon–intron boundary sequence ACAA-gtgg, indicating the partial absence of Exon17 and the skipping of Exon18 (Figure 2D). This boundary was the same as predicted by the MutationTaster.

### Effect of c.2395G>A Mutation on Alternative Splicing *in vitro*

**FIGURE 3:**
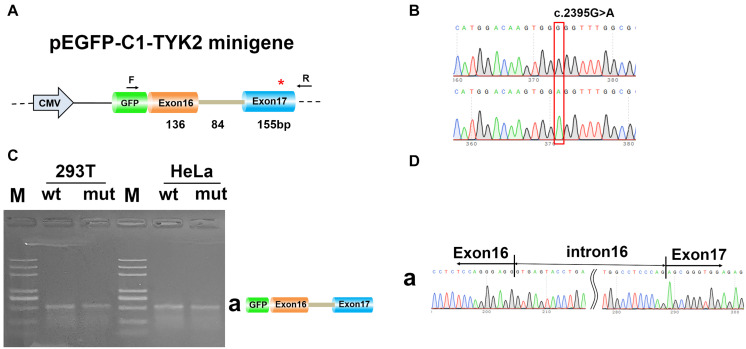
Minigene analysis based on pEGFP-C1-TYK2-wt/mut recombinant vector. **(A)** Schematic illustration of cloned vectors. **(B)** Sanger sequencing results of the recombinant vector. **(C)** Electrophoresis results of transcript PCR products in both 293T and HeLa cell lines. **(D)** Sanger sequencing of PCR products. Red * indicates the mutation site.

**FIGURE 4:**
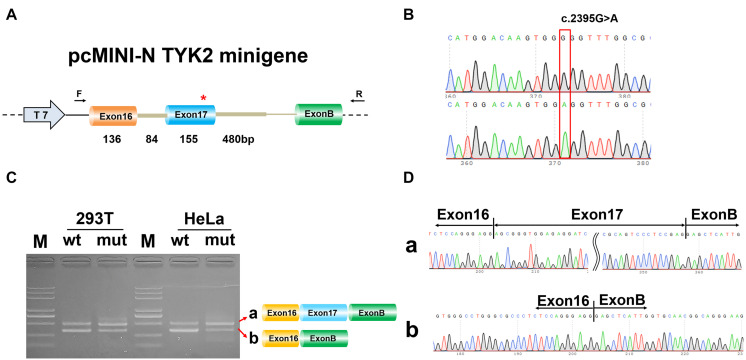
Minigene analysis based on pcMINI-N-TYK2-wt/mut recombinant vector. **(A)** Schematic illustration of cloned vectors. **(B)** Sanger sequencing results of the recombinant vector. **(C)** Electrophoresis results of transcript PCR products in both 293T and HeLa cell lines. **(D)** Sanger sequencing of PCR products. Red *indicates the mutation site.

To determine whether c.2395G>A disrupted splicing of TYK2, the entire genomic region spanning from TYK2 Exon16 to Exon17 was cloned into pEGFP-C1-TYK2-wt/mut and expressed as minigenes in human 293T and HeLa cells. The results showed that there is only one PCR band with the same size for both WT and mut type in both 293T and HeLa (Figure 3). The Sanger sequencing results showed that this band was Exon16 (136 bp)–Intron16 (84 bp)–Exon17 (155 bp); no other forms of splicing occurred (Figure 3). In addition, the complete Exon16 (136 bp), Exon17 (155 bp), Intron16 (84 bp), and part of Intron17 (480 bp) sequences were inserted into the pcMINI-N vector to construct the pcMINI-N-TYK2-wt/mut recombinant vector, which contained a universal MSC-IntronB-ExonB sequence. The PCR results showed two PCR bands (bands a and b, Figure 4). Sequencing results showed that band a was a regular splicing band [Exon16 (136 bp)–Exon17 (155 bp)–ExonB, Figure 4]; band b spliced the complete Exon17 [Exon16 (136 bp)–ExonB, Figure 4]. In summary, there was no difference between the WT and the mut type in the two minigene construction strategies, indicating that mutation c.2395G>A did not affect splicing *in vitro*.

## Discussion

TYK2 is one of the JAK protein tyrosine kinase family members that associate with the receptors like the type 1 IFN receptor (IFNAR), IL-10 family receptors (IL-10R and IL-22R), and IL-12 family receptors (IL-12R and IL-23R) (Strobl et al., 2011). People naturally deficient in TYK2 have increased sensitivity to mycobacterial and viral infections (Kreins et al., 2015; Fuchs et al., 2016). Based on this patient, our previous studies found that the expression levels of Tyk2 protein and gene were decreased by c.2395G>A mutation (Wu et al., 2020), so his susceptibility to mycobacterial and viral infections was well explained. This mutation caused the decrease of protein content and caused the occurrence of variable splicing in the patient. We found that the substitution amino acid induced by c.2395G>A does alter *TYK2* function through bioinformatics prediction. According to our previous study (Wu et al., 2020), there was no expressed TYK2 protein detected in the patient with homozygous mutation, indicating that it would be loss-of-function, in which case the patient is likely ill because of homozygosity for the missense protein. In addition, the expression of band a in the patient was about 70% of that in the parents, but no TYK2 protein was detected, indicating that the possible cause of the disease is due to the substitution of amino acids, which might lead to protein inactivation or incomplete synthesis. We did not detect band b in the parental samples, which might be caused by the dominant effect or epistatic effect of alleles. As we know, most genetic traits are much more complex than a simple dominant/recessive relationship; therefore, there might be other ways to inhibit the expression of splicing in heterozygotes. Hence, more experiments were needed to reveal the mechanism. For the splicing of *TYK2*, the actual variable splicing start site is consistent with that predicted by the MutationTaster, indicating that it is a donor site. This alternative splicing pattern complied with the GT-AG rule (Burset et al., 2000). Previous reports showed that alternations near intron boundaries or cis-splicing regulatory elements could change the efficiency or patterns of splicing (Lee and Rio, 2015; Ramanouskaya and Grinev, 2017). The consensus sequence is ACAA/gtgg, with the GT after CAA as the donor site, similar to Ohno’s reports (Ohno et al., 2018). The last two bases of exons are highly conserved as AG in 80% of 50 splice sites (Demirci et al., 2004). This mutation in our present results could probably disrupt the exon–intron junction.

However, the puzzle is that there was no difference between the WT and the mut type in the two minigene construction strategies. Combined with the results of *in vivo*, we speculated that the effect of the mutation on splicing was not absolute but instead in degree. According to the gray scale of the two PCR bands, this site might have a weak influence on splicing. So this may be the reason for the negative result of minigene *in vitro*. Due to the limitation of minigene technology, it is impossible to insert the whole sequence from Exon17 to Exon19 (the sequence is too long to insert), which might be the fundamental reason for the absence of an alternative splicing pattern *in vitro*.

Many studies have reported on genetic variants that modulate pre-mRNA splicing and in turn influence phenotype and disease risk (Li et al., 2016; Manning and Cooper, 2017; Park et al., 2018). These studies have a wide range of implications, because they can reveal the pathogenesis of diseases and provide information for the treatment options of diseases (Nielsen et al., 2007; Gregory et al., 2012; Ng et al., 2012; Matesanz et al., 2015). Pre-mRNA splicing is a highly regulated process. Each exon is controlled by a combination of flanking splicing sites and multiple splicing regulatory elements. These elements can exist in exon and intron sequences and are not easy to be identified by sequence (Li et al., 2020). Usually, they act as binding platforms for splicing regulators (such as Tyk2 protein) and can promote exon inclusion (enhancer) or cause exon jumping (silencer).

## Data Availability Statement

The data presented in the study are deposited in the Genome Sequence Archive for Human (GSA-Human) repository, accession number HRA000803.

## Ethics Statement

The studies involving human participants were reviewed and approved by the Ethics Committee of The First Affiliated Hospital of Fujian Medical University [ID: MRCTA, ECFAH of FMU (2019)218]. Written informed consent to participate in this study was provided by the participants’ legal guardian/next of kin.

## Author Contributions

SC and PW substantially contributed to the conception and design of the work, drafted the work and revised it critically for important intellectual content. BW and CL completed the clinical examination and sample collection. LC, CL, JC, and GL helped perform the experiments and analyzed the data. BW and SC involved in the analysis and interpretation of data for the work. PW agreed to be accountable for the work in ensuring that questions related to the integrity of any part of the work are appropriately investigated and resolved. All authors read and approved the final manuscript.

## Conflict of Interest

The authors declare that the research was conducted in the absence of any commercial or financial relationships that could be construed as a potential conflict of interest.

## Footnotes

1 www.umd.be/hsf3

2 http://www.mutationtaster.org

3 https://spliceailookup.broadinstitute.org

4 https://github.com/VCCRI/Spliceogen

5 https://github.com/gagneurlab/MMSplice

## Supplementary Material

Supplementary Table 1 Primer sequence information.

Supplementary Table 2 Database prediction information.
