# Supplementary material for: A Missense Mutation rs781536408 (c.2395G>A) of TYK2 Affects Splicing and Causes Skipping of Exon18 in vivo
Source: Front Genet. 2021 Jun 21;12:679678. doi: 10.3389/fgene.2021.679678 (PMC8255812; doi:10.3389/fgene.2021.679678)
Supplement: Supplementary Table 1 — Primer sequence information. [file Table_1.DOCX]

Table S1 Primer sequence information

| **Primer ID** | | **Sequence (5’- -3’)** |
| --- | --- | --- |
| MinigenePrimers | 21716-TYK2-F | cttctgacctcgtgatccatcc |
|  | 22119-TYK2-F | gaatcagctaggctgtggtgatg |
|  | 23778-TYK2-R | ccccagagtggaccgccaggtgc |
|  | 24150-TYK2-R | cagggcatgcttatgaatgc |
|  | GFP-TYK2-EcoR1-F | CTTCGAATTCcGAGAACAAGAACCTGGTTCATGG |
|  | GFP-TYK2-Kpn1-R | CCCGCGGTACCCTCGGAGGGACTGCGGCTCTGC |
|  | TYK2-mut-F | CCATGGACAAGTGGAGGTTTGGCGCCACCC |
|  | TYK2-mut-R | GGGTGGCGCCAAACCTCCACTTGTCCATGG |
|  | MINI-N-TYK2-Kpn1-F | GCTTGGTACCATGGAGAACAAGAACCTGGTTCATG |
|  | MINI-N-TYK2-EcoR1-R | TGCAGAATTCgaggctgagacaggagaatcgc |
| qRT-PCR Primers | TYK2-qpcr-1F | CAGATCAGACAGCACAGGGG |
|  | TYK2-qpcr-1R | GCAGTCCTTGAAGCTGGTCT |
|  | TYK2-qpcr-2F | TCAAGATCGGGGACTTTGGC |
|  | TYK2-qpcr-2R | ATCTGACCCTGAGCAATGCC |
|  | TYK2-qpcr-band a-F | TCAAGCTGAGTGATCCTGGC |
|  | TYK2-qpcr-band a-R | ACCTCCACTTGTCCATGGCGG |
|  | TYK2-qpcr-band b-F | TCAAGCTGAGTGATCCTGGC |
|  | TYK2-qpcr-band b-R | CAGCAAGATTGTCCATGGCG |
| In vivo PCR primers | TYK2-E13-F | GACCCTGAGGAGGGCAAGATGGATG |
|  | TYK2-E14-F | CACGCACCTGGCCTTCGTGCATG |
|  | TYK2-E19-R | CTTGTGGAAAACCGTAGGGTCCG |
|  | TYK2-E21-R | GTCTCGGAGGCTGCCCAGGGGC |
